# Prognostic Value of MicroRNA-182 in Cancers: A Meta-Analysis

**DOI:** 10.1155/2015/482146

**Published:** 2015-05-07

**Authors:** Fei Wang, Shanliang Zhong, Haijun Zhang, Wei Zhang, Hongming Zhang, Xue Wu, Baoan Chen

**Affiliations:** ^1^Department of Hematology (Key Department of Jiangsu Medicine), Zhongda Hospital, Medical School, Southeast University, Nanjing 210009, China; ^2^Center of Clinical Laboratory Science, Jiangsu Cancer Hospital Affiliated to Nanjing Medical University, Nanjing 210009, China; ^3^Department of Oncology, Zhongda Hospital, Medical School, Southeast University, Nanjing 210009, China; ^4^Department of Pathology, Medical School, Southeast University, Nanjing 210009, China

## Abstract

*Objective*. MicroRNA-182 (miR-182) exhibits altered expression in various cancers. The aim of this study was to investigate the predictive value of miR-182 expression for cancer patient survival. *Methods*. Eligible studies were identified through multiple search strategies, and the hazard ratios (HRs) for patient outcomes were extracted and estimated. A meta-analysis was performed to evaluate the prognostic value of miR-182. *Results*. In total, 14 studies were included. A high miR-182 expression level predicted a worse outcome with a pooled HR of 2.18 (95% CI: 1.53–3.11) in ten studies related to overall survival (OS), especially in Chinese populations. The results of seven studies evaluating disease-free survival/relapse-free survival/recurrence-free interval/disease-specific survival (DFS/RFS/RFI/DSS) produced a pooled HR of 1.77 (95% CI: 0.91–3.43), which was not statistically significant; however, the trend was positive. When disregarding the DSS from one study, the expression of miR-182 was significantly correlated with DFS/RFS/RFI (pooled HR = 2.52, 95% CI: 1.67–3.79). *Conclusions*. High miR-182 expression is associated with poor OS and DFS/RFS/RFI in some types of cancers, and miR-182 may be a useful prognostic biomarker for predicting cancer prognosis. However, given the current insufficient relevant data, further clinical studies are needed.

## 1. Introduction

Cancer is a major public health challenge in many parts of the world. Although overall cancer mortality decreased by 20% between 1991 and 2010, cancer remains one of the most common causes of death worldwide [1]. Early detection and precise diagnosis are critical for these patients.

MicroRNAs (miRNAs), which are approximately 22 nucleotides in length, bind to complementary sequences of mRNA at the 3′-untranslated region and result in the downregulation of protein-coding target genes in the nucleus and cytoplasm. miRNAs play important roles in various biological processes, such as cellular growth, development, differentiation, proliferation, apoptosis, angiogenesis, and metastasis [2, 3]. The aberrant expression of miRNAs has been observed in various diseases, including human carcinomas [4]. Many studies have demonstrated that oncogenic miRNAs are frequently upregulated in tumors, whereas tumor-suppressive miRNAs are frequently downregulated. Therefore, the success of utilizing miRNAs as diagnostic or prognostic biomarkers has received substantial attention in cancer research [5].

miR-182, a member of the miR-183 family located on 7q31-34, is one of the most frequently studied cancer-related oncogenic miRNAs that is dysregulated in various cancer tissues [6]. Several studies have reported that miR-182 is abnormally expressed in many cancer types. Furthermore, miR-182 may function as an oncogenic miRNA to enhance cancer cell proliferation, survival, aggressiveness, tumorigenesis, and drug resistance [7–9]. However, other studies have reported that miR-182 is downregulated in gastric adenocarcinoma and that increased miR-182 levels are correlated with clinical treatment benefits [10, 11]. Together, these results suggest a highly complex mechanism of miR-182-related tumorigenesis.

Several studies have recently suggested that upregulated miR-182 expression is significantly associated with poor cancer prognosis; other studies have reported conflicting results. Therefore, to assess the cumulative evidence regarding the possible association between elevated miR-182 and poor outcomes in cancer patients and to explore the possibility of miR-182 as a prognostic biomarker, we conducted a meta-analysis of relevant studies. Through this study, we aim to clarify the role of miR-182 in human carcinomas and to investigate this association.

## 2. Materials and Methods

### 2.1. Search Strategy

To identify eligible studies, we performed an online search using PubMed and the Web of Science through December 31, 2014. The search strategy employed terms related to miR-182 (e.g., “miR-182,” “mircoRNA-182,” and “microRNA182”). A manual review of the references of relevant publications was also carefully performed to obtain additional information. All searches were conducted independently by two reviewers, and the differences were checked and resolved by discussion.

### 2.2. Inclusion and Exclusion Criteria

Studies were eligible if they met the following criteria: (1) the study subjects were patients with any type of cancer; (2) miR-182 expression was measured in tumor tissue or plasma; (3) the relationship between miR-182 expression and clinical outcomes was reported; and (4) the full-text article was available in English. Studies were excluded based on the following criteria: (1) reviews, letters, or laboratory reports; (2) overlapping or duplicate data; (3) studies of nondichotomous miR-182 expression levels; and (4) the absence of key information regarding survival outcome, such as hazard ratios (HRs) or 95% confidence intervals (CIs) or no way to calculate such parameters.

### 2.3. Data Extraction

Two investigators evaluated and extracted the data independently from all eligible studies under the guideline of a critical review checklist from the Dutch Cochrane Centre proposal for the meta-analysis of observational studies in epidemiology (MOOSE) [12]. The following items were extracted: first author, year of publication, country of origin, tumor type, sample type and number, method, cutoff value, follow-up and HRs of miR-182 for overall survival (OS), and the corresponding 95% CIs. If not available, data were calculated following Tierney et al.'s method [13]. Disagreements were resolved by discussion. All decisions regarding the data were resolved by consensus.

### 2.4. Statistical Analysis

All of the HRs and their corresponding 95% CIs were used to calculate the pooled HR. Generally, if the overall HR was >1 and if the 95% CI did not overlap in the forest plot, high miR-182 expression was considered to be significantly associated with poor survival rate. Cochran's *Q* test and Higgins' *I* ^2^ statistic were used to assess heterogeneity. A *P* < 0.10 or *I* ^2^ > 50% suggested significant heterogeneity in the literature and a random-effect model was used; otherwise, a fixed-effect model was used. Begg's funnel plot and Egger's test were used to evaluate the potential publication bias among the studies. *P* < 0.05 was considered significant, and all *P* values were two-sided. All analyses were performed using Stata 12.0 (Stata Corporation, College Station, TX, USA) and Review Manager 5.3 (Cochrane Collaboration, Oxford, UK).

## 3. Results

### 3.1. Summary of Included Studies

**Figure 1:**
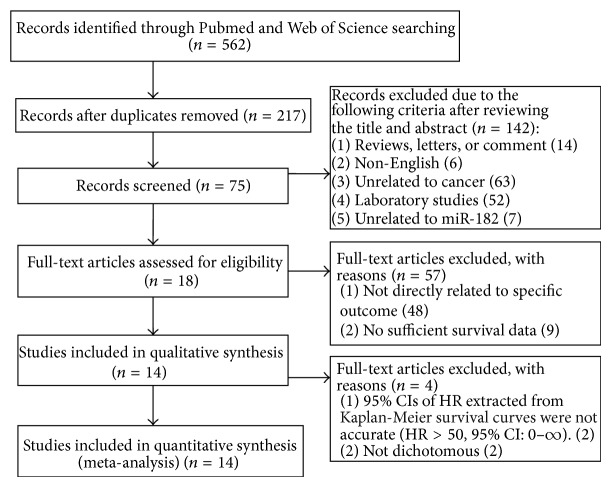
Flow diagram of the study selection process.

In total, 562 studies on miR-182 were collected in our initial search. 345 studies were removed because of duplication, and 142 studies were excluded after manually screening the titles and abstracts. After reading the full-texts of the remaining 75 studies, 57 were excluded. Finally, 18 studies [7, 11, 14–29] evaluating the relationship between miR-182 expression and cancer patient survival were selected and are listed in Table 1. After further screening, we determined that the survival data of two articles could not be applied and that the data of an additional two articles were not dichotomous. Therefore, we included 14 eligible studies in the final meta-analysis (Figure 1).

Of the 14 included studies, 943 participants with OS data and 777 participants with disease-free survival/relapse-free survival/recurrence-free interval/disease-specific survival (DFS/RFS/RFI/DSS) data from China, the USA, Germany, France, Greece, Norway, and India were analyzed. The types of malignant cancers included colorectal cancer (*n* = 3), nonsmall cell lung cancer (NSCLC) (*n* = 2), pancreatic cancer (*n* = 2), prostate cancer, ovarian cancer, medulloblastomas, glioma, breast cancer, muscle-invasive bladder cancer (MIBC), and hepatocellular carcinoma (HCC). Fresh, frozen or formalin-fixed and paraffin-embedded (FFPE) tissues were used in 13 studies, whereas plasma was used in one study. Quantitative real-time PCR (qRT-PCR) was used in 11 studies, and in situ hybridization (ISH) was used in the remaining 3 studies.

Among these studies, seven articles evaluated OS, four articles evaluated DFS/RFS/RFI/DSS, and three studies evaluated both OS and DFS/RFS. Nine studies directly reported HRs and 95% CIs. Only one study reported RR; therefore, we combined HR and RR [30]. We calculated HRs from survival curves in four studies.

### 3.2. Overall Survival Is Associated with miR-182 Expression

**Figure 2:**
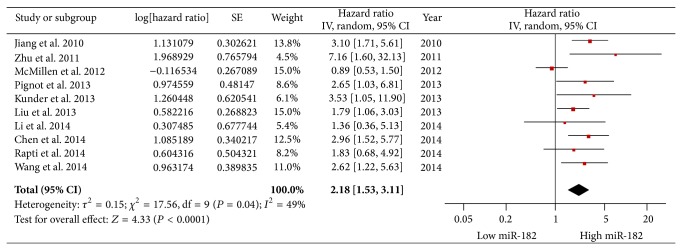
Forest plots of studies evaluating the hazard ratios of high and low miR-182 expression with respect to overall survival.

Ten articles evaluated OS for miR-182, and significant heterogeneity between studies was found (*P* = 0.04, *I* ^2^ = 48.8%); therefore, a random-effects model was applied. Our results revealed that high miR-182 expression predicted worse outcomes with a combined HR of 2.18 (95% CI: 1.53–3.11) (Figure 2).

**Figure 3:**
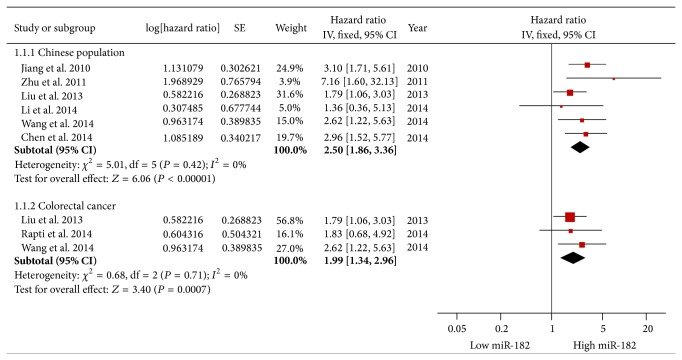
Subgroup analysis of overall survival in Chinese or colorectal cancer patients.

Considering the large proportion of Chinese patients in the studies, we performed a stratified analysis by classifying studies into subgroups of ethnicity. The Chinese subgroup exhibited a better association between elevated miR-182 expression and poor OS (pooled HR = 2.50, 95% CI: 1.86–3.36) via a fixed-effects model (*P* = 0.42, *I* ^2^ = 0%) (Figure 3).

Among the ten studies related to OS, three involved colorectal cancer. Therefore, we performed a corresponding subgroup analysis that revealed a correlation between elevated miR-182 and worse OS in colorectal cancer (pooled HR = 1.99, 95% CI: 1.34–2.96) via a fixed-effects model (*P* = 0.71, *I* ^2^ = 0%) (Figure 3).

### 3.3. Tumor Progression Is Associated with miR-182 Expression

**Figure 4:**
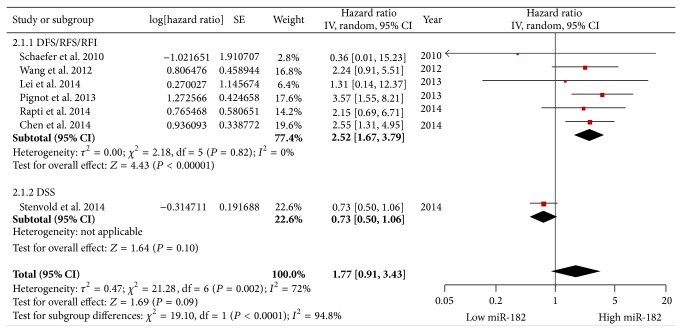
Forest plots of studies evaluating the hazard ratios of high and low miR-182 expression with respect to DFS/RFS/RFI/DSS.

A fixed-effects model (*P* = 0.82, *I* ^2^ = 0%) was also used for studies evaluating DFS/RFS/RFI. The expression of miR-182 was significantly correlated with DFS/RFS/RFI (pooled HR = 2.52, 95% CI: 1.67–3.79). However, elevated miR-182 levels were not significantly correlated with the data combining DFS/RFS/RFI with DSS (pooled HR = 1.77, 95% CI: 0.91–3.43) using a random-effects model (*P* = 0.002, *I* ^2^ = 72%); however, the trend was positive (Figure 4).

### 3.4. Publication Bias

**Figure 5:**
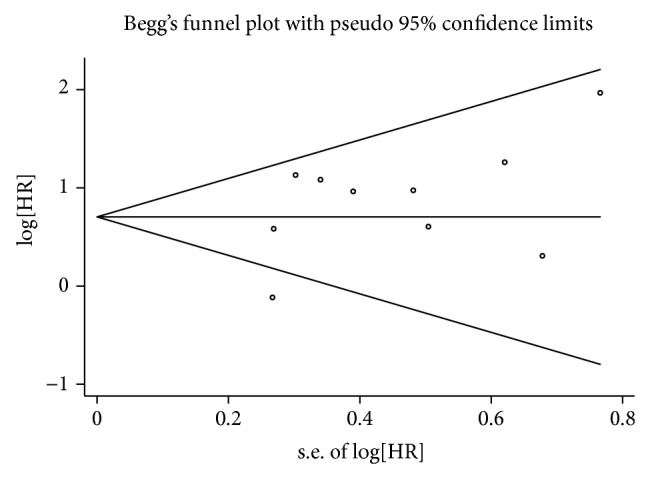
Begg's funnel plots for publication bias in overall patient survival.

**Figure 6:**
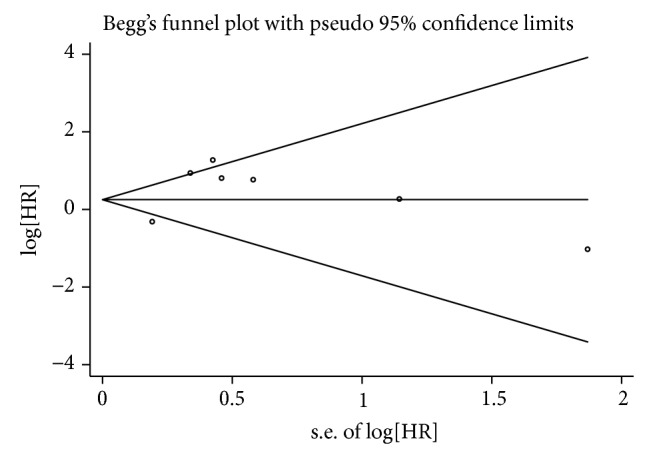
Begg's funnel plots for publication bias with respect to DFS/RFS/RFI/DSS.

Begg's funnel plots and Egger's tests were used to evaluate the publication bias of all studies regarding patient survival and tumor progression. As shown in Figures 5 and 6, most of the funnel plots were symmetrical, and the *P* values of Egger's test were 0.188 for OS and 0.320 for DFS/RFS/RFI/DSS, suggesting the absence of significant publication bias.

## 4. Discussion

In recent years, miRNAs have attracted increasing interest among investigators, particularly cancer researchers, as vital cellular molecules involved in normal and pathological states. Many studies have demonstrated that miRNAs are aberrantly expressed in different classes of cancers and can be used as novel biomarkers of tumor identification and prognosis [5, 31–33]. Among these miRNAs, miR-182 (which belongs to the miR-183-96-182 cluster) is considered a microoncogene. Extensive profiling studies over the past several years have linked the dysregulated expression of miR-182 to several cancer types, including colorectal cancer, lung cancer, glioma, bladder cancer, endometrial carcinoma, prostate cancer, and ovarian cancer [14, 15, 20, 34].

miR-182 is involved in several key steps of tumorigenesis, including epithelial-mesenchymal transition, cell cycle regulation, proliferation, survival, migration, aggressiveness, and drug resistance [7–9, 35]. miR-182 plays a crucial role in tumorigenesis and progression, and miR-182 may become a potential therapeutic target and biomarker of tumor diagnosis and prognosis [36]. To our knowledge, there is no meta-analysis investigating the associations between miR-182 expression and the prognosis of various cancers. Therefore, we gathered the available evidence from all relevant studies to evaluate the prognostic values of miR-182.

In our study, increased expression of miR-182 was found to predict poor survival in patients with a variety of cancers. The combined HR of OS was 2.18 (95% CI: 1.53–3.11), indicating that elevated miR-182 levels are closely linked to the prognosis of patients with malignancies. This was particularly true in the Chinese cancer population, for which the pooled HR for OS was 2.5 (95% CI: 1.86–3.36), further demonstrating the predictive value of miR-182. Our stratified analysis suggested a closer relationship between rising miR-182 levels and poor survival in the Chinese subgroup. Among ten studies reporting on OS in seven tumor types, three were related to colorectal cancer. Therefore, we performed a subgroup analysis of colorectal cancer. The result also revealed that elevated miR-182 yielded worse OS in colorectal cancer (pooled HR = 1.99, 95% CI: 1.34–2.96). Due to the limited number of eligible studies for each cancer type, further research is needed to determine whether pathological cancer types impact the prognostic role of miR-182.

Because the included studies used a variety of indices to evaluate tumor progression, such as DFS, RFS, RFI, and DSS, we combined these indices to evaluate the prognostic value of miR-182. The results did not indicate an obvious association between high miR-182 expression and DFS/RFS/RFI/DSS (pooled HR = 1.77, 95% CI: 0.91–3.43); however, the trend was positive. After excluding the DSS reported in one study, the expression of miR-182 was significantly correlated with DFS/RFS/RFI (pooled HR = 2.52, 95% CI: 1.67–3.79). Because the HR of DSS from one study was 0.73 (95% CI: 0.50–1.06), marked heterogeneity was observed in the DFS/RFS/RFI/DSS group. The author [15] reported that there was a tendency towards a better prognosis for NSCLC patients overexpressing miR-182. This heterogeneity with respect to other studies may be attributed to the differences in the types of cancer, methods of detection, or miR-182 cutoff values. Alternatively, high miR-182 expression may be a favorable prognostic factor in NSCLC. Therefore, further study is needed to confirm the role of miR-182 in predicting the prognosis of different cancer types.

The overexpression of miR-182 has been shown in many studies to be associated with poor outcomes in several cancer types, and our results support these conclusions. However, why miR-182 is associated with poor prognosis in many cancers remains poorly understood. Recent studies have reported several underlying mechanisms that may play key roles, especially in metastasis. Accumulating evidence suggests that miR-182 regulates tumor cell invasion and metastasis. Sachdeva et al. [37] identified miR-182 as an overexpressed miRNA in a subset of soft tissue sarcomas that metastasized to the lungs in a mouse model and demonstrated that miR-182 was a driver of tumor metastasis in vivo by enhancing the activation of extracellular proteases, including urokinase and MMP-9, and repressing multiple proteins that prevent tumor cell intravasation. In a clinical analysis, Pignot et al. reported upregulated miR-182 in both muscle-invasive bladder cancer (MIBC) and non-muscle-invasive bladder cancer (NMIBC) patients; furthermore, miR-182 was found to be related to the aggressiveness of MIBC tumors [7]. However, Yang et al. reported that miR-182 upregulation could inhibit metastatic activity by silencing FOXO3 expression that suggested that miR-182 may function as an oncogenic miRNA for lung cancer growth as well as a suppressor of lung cancer metastasis [38]. Kong et al. demonstrated that miR-182 targeted the cAMP-responsive element binding protein 1 (CREB1) gene and suppressed gastric adenocarcinoma cell growth [10]. These results indicate that miR-182 plays a pivotal role in carcinogenesis, possibly with different mechanisms in various cancer subtypes.

Irrespective of the mechanism or clinical verification of miR-182, the results suggest that miR-182 can be used as a predictive biomarker of cancer prognosis. However, we make this conclusion cautiously, and some details must be further refined for several reasons. First, the reliability of our results is questionable in light of the number of eligible studies for OS and DFS/RFS/RFI. Additionally, the patient populations were limited to Asia, Europe, and the USA, lacking data from other regions, which might impact the statistical power of analysis. Therefore, our results need to be confirmed by more studies with larger sample sizes and other regions. Second, some HRs were extracted and calculated from survival curves, which may lead to small errors, and the HRs extracted from survival curves are univariate analysis; when possible, pooled HRs should be presented based on multivariate analysis. Third, although no significant publication bias was detected in our study, language bias may have been present because of the restriction to English. Additionally, different detection methods, sample sources, and cutoff values may also have affected the effectiveness of miR-182 as a predictive biomarker of prognosis, causing relatively large heterogeneity; however, it is impossible to include only studies with uniform features due to limited sample size. Forth, our study found high miR-182 expression to play a prognostic role in various cancers; however, it is impossible to confirm that miR-182 is an independent predictive factor based on our results. Finally, all included studies were retrospective, which may weaken the values of pooled results.

In conclusion, despite the limitations described above, our findings clearly indicate that high miR-182 expression is significantly associated with poor OS and DFS/RFS/RFI and may be a suitable prognostic biomarker in some cancer types, especially in Chinese populations. However, the current data are insufficient. These findings must be confirmed in further multicenter prospective clinical studies.

## Figures and Tables

**Table 1:** Characteristics of the studies included in the meta-analysis.

Author	Year	Country	Type	Sample	Number	Method	Cutoff	Survival	HR	Max follow-up (months)
Chen	2014	China	Pancreatic	Plasma	109	qRT-PCR	Youden index	OS, DFS	R	60
Rapti	2014	Greece	Colorectal	Frozen tissue	105 OS	qRT-PCR	65%	OS, DFS	R	120
					92 DFS					
Wang	2014	China	Colorectal	FFPE	138	ISH	Score = 3 (0–7)	OS	R	96
Li	2014	China	Pancreatic	Primary tissue	38	qRT-PCR	Matched nontumor tissue	OS	SC	36
Pignot	2013	France	MIBC	Frozen tissue	72	qRT-PCR	NR	OS, RFS	SC	65
Kunder	2013	India	Group 3 and 4 medulloblastomas	Frozen tissue + FFPE	37	qRT-PCR	Log_2_ (relative quantity)	OS	R	60
Liu	2013	China	Colorectal	Frozen tissue	148	qRT-PCR	Median	OS	R	89
McMillen	2012	USA	Grade III or IV ovarian	FFPE	107	ISH	Score > 1.5 (0–3)	OS	R	82
Zhu	2011	China	NSCLC	Frozen tissue	70	qRT-PCR	Median	OS	R	25
Jiang	2010	China	Glioma	PE	119	qRT-PCR	Median	OS	(RR) R	80
Stenvold	2014	Norway	NSCLC	FFPE	305	ISH	Score > 0 (0–3)	DSS	R	234
Lei	2014	China	Breast	Fresh tumor specimens	38	qRT-PCR	Median	RFS	SC	50
Wang	2012	China	HCC	Frozen tissue	86	qRT-PCR	Median	DFS	SC	56
Schaefer	2010	Germany	Prostate	Frozen tissue	75	qRT-PCR	Median	RFI	R	93
Casanova-Salas^a^	2014	Spain	Prostate	FFPE	272	qRT-PCR	Low/medium/high	PFS	SC	189
Rodríguez-González^a^	2011	Netherlands	ER+ breast	Tissue	246	qRT-PCR	Divided into quarters	PFS	SC	24
Hirata^b^	2012	USA	Bladder	FFPE	18	qRT-PCR	Median	OS	SC	120
Hirata^b^	2013	USA	Prostate	FFPE	52	qRT-PCR	Median	OS	SC	100

DFS: disease-free survival, DSS: disease-specific survival, ER: estrogen receptor, FFPE: formalin-fixed paraffin embedded tissues, HCC: hepatocellular carcinoma, HR: hazard ratio, ISH: in situ hybridization, MIBC: muscle-invasive bladder cancer, NR: not reported, NSCLC: nonsmall lung cancer, OS: overall survival, PFS: progression-free survival, R: reported, RFI: recurrence-free interval, RFS: relapse-free survival, RR: relative risk, and SC: survival curve.

^a^Excluded due to nondichotomy; ^b^95% CIs of HR extracted from the Kaplan-Meier survival curves were not accurate (HR > 50, 95% CI: 0–∞).
